# Supplementary material for: A specific class of infectious agents isolated from bovine serum and dairy products and peritumoral colon cancer tissue
Source: Emerg Microbes Infect. 2019 Aug 14;8(1):1205–18. doi: 10.1080/22221751.2019.1651620 (PMC6713099; doi:10.1080/22221751.2019.1651620)
Supplement: Supplemental Material [file TEMI_A_1651620_SM3013.zip › suppl_data/BMMF2_paper_20.7_table_sup.docx]

**A Specific Class of Infectious Agents Isolated from Bovine Serum and Dairy products and Peritumoral Colon Cancer Tissue**

Ethel-Michele de Villiers^1,*^, Karin Gunst^1^, Deblina Chakraborty^1^, Claudia Ernst^1^, Timo Bund^1^ and Harald zur Hausen^1^

^1^ - Episomal-Persistent DNA in Cancer- and Chronic Diseases, Deutsches Krebsforschungszentrum, 69120 Heidelberg, Germany

^*^ - Corresponding author:

Ethel-Michele de Villiers

address: Deutsches Krebsforschungszentrum

Im Neuenheimer Feld 280

69120 Heidelberg

Germany

e-mail: [e.devilliers@dkfz.de](mailto:e.devilliers@dkfz.de)

telephone: +49 6221 423850

Orcid 0000-0003-4357-0567

e-mail: Karin Gunst [k.gunst@dkfz.de](mailto:k.gunst@dkfz.de)

Deblina Chakraborty d.chakraborty@dkfz.de ORCID 0000-0002-3755-6809

Claudia Ernst claudia.ernst@dkfz.de

Timo Bund [t.bund@dkfz.de](mailto:t.bund@dkfz.de) ORCID 0000-0002-3304-4494

Harald zur Hausen [zurhausen@dkfz.de](mailto:zurhausen@dkfz.de) ORCID 0000-003-1103-0347

Abstract:

The *in silico* analyses of 109 replication competent genomic DNA sequences isolated from cow milk and its products (97 in the bovine meat and milk factors 2 group – BMMF2, and additional 4 in BMMF1) seems to place these in a specific class of infectious agents spanning between bacterial plasmid and circular ssDNA viruses. Satellite-type small plasmids with partial homology to larger genomes, were also isolated in both groups. A member of the BMMF1 group H1MBS.1 was recovered in a distinctly modified form from colon tissue by laser microdissection. Although the evolutionary origin is unknown, it draws the attention to the existence of a hitherto unrecognized, broad spectrum of potential pathogens. Indirect hints to the origin and structure of our isolates, as well as to their replicative behavior, result from parallels drawn to the Hepatitis deltavirus genome structure and replication.

**Key words**: infectious bovine meat milk factors recover colon tissue

**Introduction:**

A large set of novel viral and phage genomes has been identified during the past several years. These were identified through analyses of the human microbiome [1,2] or of samples from other sources. Metagenomic analyses are commonly applied [3,4,5], although the application of more conservative techniques also led to the identification of a number of new infectious agents [6,7,8]. Analyses mostly relied on comparisons to known agents or the identification of a replication gene in the assembled genome. Unfortunately, several studies concentrated only on the presence of known infectious entities, while paying less attention to identifying yet unknown infectious agents [9,10]. One difficulty originates from the discovery of chimeric genomes. This weakened the strict demarcation between certain types or groups of infectious agents resulting in studies investigating their phylogenetic origin [11,12,13].

We approached the question of as yet unknown agents from a different perspective: published data on the epidemiology of colon and breast cancer suggested milk and meat factors (BMMFs) derived from dairy cattle as presumably species-specific risk for these cancers after consumption of products from these animals [8,14,15]. Therefore, we initiated studies to isolate infectious agents from bovine sera and commercially available cow milk and its products. Based on our previous experience with single stranded DNA virus genomes [6,16] we initiated experiments by gradient density fractionation of sera followed by rolling circle amplification of DNA obtained. Putative Rep genes were identified as part of the DNA sequences obtained by *in silico* comparisons to available sequences. Amplification using inverse PCR with back-to-back primers in the rep gene led to the isolation of full and partial circular DNA genomes from bovine sera [17].

This was extended to samples from commercially available cow milk for the presence of specific circular single-stranded DNA genomes. Four additional isolates were obtained from human brain and serum (from patients with multiple sclerosis). A total of 18 full-length circular single-stranded DNA molecules (~1100 to 3000 nucleotides) were cloned and sequenced [17,18,19,20]. We divided the initial 18 isolates into four different groups BMMF1 through BMMF4, according to their molecular characteristics [14]. Three of these groups revealed a remarkable degree of similarity to *Acinetobacter baumannii* and Psychrobacter plasmids. The fourth group comprised 3 isolates being representatives of *Genomoviridae (Gemycircularvirus)*.

The isolation of these circular DNA molecules, in part closely related to bacterial plasmids, raised the question whether they represented bacterial contaminants or whether these were bacteria-related sequences adapted to infect and replicate in vertebrate and human cell. Presently representatives of all four groups have been analyzed for expression of genetic activity and replication in human cells [14,21].

We further extended our search for additional DNA plasmids in the BMMF1 and BMMF2 groups in the present study. We also describe the detection by mass spec of peptides specific for 3 new BMMF2 isolates, as well as the recovery of a modified BMMF1 isolate H1MSB.1, from colon tissue. The intention of this communication is to draw the attention to the existence of a hitherto unrecognized, broad spectrum of potential pathogens.

**Materials and methods:**

*Samples:* The isolates were obtained from milk (10) and dairy products (8) bought from local supermarkets. To avoid a biased selection, we chose milk in different types of containers (glass, carton, etc), and from many different providers. In addition, we tested crème fraiche, cream, curd, butter and yoghurt.

*DNA extraction*: DNA was extracted with phenol/chloroform as previously described [19]. Samples were diluted 1:1 with proteinase K buffer (0,2M Tris-HCL, pH7,5, 25mM EDTA, 0,3M NaCl, 2%SDS) and digested overnight with proteinase K (final concentration 1mg/ml, Sigma-Aldrich) at 37°C. Ethanol precipitation followed after the phenol/chloroform extraction. DNA were extracted this way from the initial 4 milk samples, whereas all other samples were subsequently subjected to an additional purification using a Nucleospin Gel and PCR Clean up Kit (Machery and Nagel) according to the manufacturer´s protocol. DNA was finally eluted in 5mM Tris-HCl, pH 8,0.

*Rolling circle amplification* was performed as described [17]. Template DNA (50ng) was incubated in a total volume of 10μl (1x phi29 DNA polymerase buffer) with 25μM Exo-resistant Random primer (Thermo Fisher Scientific) at 95°C for 3 min followed by cooling on ice. This sample was diluted to 20μl by adding BSA (0,4mg/ml), 0,75mM dNTPs (Takara) each and 10U phi29 DNA Polymerase (New England Biolabs) in 1x phi29 DNA polymerase buffer. Incubation followed at 30°C for 18h and 10 minutes at 65°C before stopping the reaction at 15°C.

*Polymerase chain reaction*: A 3μl template from the RCA amplified DNA was used in the polymerase chain reaction (PCR) using TAKARA Taq enzyme and the accompanying solutions according to the manufacturer´s protocol (LA TAKARA). The respective back-to-back primers were added at a final concentration of 0,2mM each. Primers were designed based on the conserved regions of HCBI1, HCBI2 and HCBI7 for the BMMF2 group and for the BMMF1 group based on the conserved region of the replication gene.

PCR was performed by using touchdown protocols specific for the respective primer pairs:

MBB2 primers for the BMMF1 group: Mbb1 (MSBI1.176bbF296 forward) –5´-TGCAGAAATTGCCCCTCGACT-3´ and Mbb2 (MSBI1.176bbR295 reverse) –5´-AACAATGGGGAAGAAGTCAAAGG-3´. The first round of 5 cycles amplification was performed at 30 seconds melting at 94°C, 1 minute annealing at 64°C and 2 minutes elongation at 72°C, followed by a second round of 5 cycles using 62°C annealing and a third round of 30 cycles using 60°C for annealing. The final round of 10 minutes at 72°C followed.

Amplification for the BMMF2 group was performed as described for the BMMF1 group using the MBB2 primers, except that the touchdown temperatures were 58°C, 56°C and 54°C for the LSconA primer and 56°C, 54°C and 52°C for the LSconB primer and the elongation time was prolonged to 3 minutes at 72°C per cycle. The back-to-back primer pairs used for the BMMF2 group were: LSConA5p (forward) –5´- AAGGCAGATCAACACAGG-3´ and LSconA3p (reverse) – 5´-AGCAGATTGCAAAGCCTG-3´, LSconB5p (forward) – 5´-CAACACAGGGATAGAATAAC-3´ and LSconB3p (reverse) – 5´- ATCTGCCTTAGCAGATTGC-3´. PCR products were separated by gel electrophoresis, visible fragments excised and DNA extracted using the Nucleospin Gel and PCR Clean up Kit (Machery and Nagel) according to the manufacturer´s protocol. DNA was finally eluted in 5mM Tris-HCl, pH 8,0. Eluted DNA was ligated and cloned into pCR2.1 using the TA-Cloning Kit and transformed into E.coli – all according to the manufacturer´s protocol (Invitrogen™ TA Cloning™ Kit, with pCR™2.1 Vector and One Shot™ TOP10F' Chemically Competent E. coli (Invitrogen)). Sequencing of all clones was performed by GATC. This company applies cycle sequencing technology on ABI3730XL sequencing machines. Quality Phred20 is achieved for reads up to 1100 nucleotides in length.

*Laser Dissection Microscopy (LDM):* Formalin-fixed-paraffin-embedded sections with a thickness of 8μm were mounted onto LDM-compatible membrane slides (MembraneSlide 1.0 PEN NF, Carl Zeiss) and stained with anti-Rep antibodies [15]. Antibody-stained tissue regions were specifically marked and dissected using a high intensity laser beam (PALM microbeam microscope, Carl Zeiss). The selected tissue pieces together with the underlying membrane were laser-catapulted into the caps of special opaque adhesive cap microfuge tubes (500ml, Carl Zeiss). The tissue pieces were subsequently dissolved in ATL lysis buffer (QIAamp DMA Mini Kit, Qiagen) and homogenized by pipetting before transferring into a fresh 1,5ml microfuge tube. An equal volume of Chelex resin (Chelix® 100 Resin, mesh 200-400, Bio-Rad, 5% suspension in water) was added in addition to 10μg/ml Proteinase K (Sigma), followed by incubation overnight in a thermomixer at 56°C at 750 rpm. The reaction was stopped in vortexing for 10 seconds and incubation for 8 minutes at 99°C in a thermomixer. Chelex beads were removed by centrifugation and the DNA containing supernatant collected for further processing. RCA was performed as described above, followed by PCR amplification using NnXn or NoXo back-to-back primers as previously described [19]. Products were cloned and sequenced as described above.

*In silico analyses:* All sequences obtained were compared to nucleotide sequences available in Genbank by using BLASTN and the putative proteins to BLASTP (swissprot and swissprot_splicevar). In addition, Domainsweep [22] was used to identify the domain architecture within a protein sequence by using different database searches including blockssearcher, cddsearch, cathscan, pfamascan, printscan, prodomblast, prosite, smartscan, superfamily and tigrfamscan.
Predicted genes were obtained with an in-house ORFmap program, as well as with the gene identification program GENSCAN which was developed by Chris Burge from Stanford University [23]. It analyzes genomic DNA sequences from human, other vertebrates, invertebrates and plants. Other more sensitive prediction tools (Augustus [24] or Genemark [25]), were considered not suitable for our purposes, as they rely on calibration with species-specific training sets. Genscan was chosen because information is provided on the presence of polyA signals and our isolates [21] had been shown to replicate in human cells.

*Phylogenetic trees* were developed using maximum likelihood based on aligned (MUSCLE algorithm) sequences - both full-length nucleotide genomes, as well as the core region of Rep. Phylogenetic and molecular evolutionary analyses were conducted using MEGA version X [26].

Data availability*:* Genome sequencing files are available at the European nucleotide archive (<https://www.ebi.ac.uk/ena>) under Project PRJEB30101. Accession numbers LR215494-LR215600 were allocated for the respective sequences (re Table 1).

**Results:**

***In silico characterization of additional BMMF1 (Bovine Meat and Milk Factor) isolates:***

The BMMF1 group of isolates consists of 13 circular DNA genomes [14,19,27] (Suppl. Table 1). Mutual characteristics included the presence of an initiator replication protein RepB, an iteron-like tandem repeat region (3 x 22nt plus a partial repeat of 17 /18nt, in one isolate 4 complete tandem repeats) and a conserved palindromic structure located upstream of the repeats which may serve as a putative origin of replication. The RepB protein and the iterons were similar to those found in bacterial plasmids, whereas the putative origin of replication structure was similar to the nonanucleotide stem-loop origin of replication in single-stranded plant and animal viruses [3,14]. The genomes of all the isolates shared nucleotide identity to various plasmids of *Acinetobacter baumanii* and one isolate HCBI5.173 nucleotide identity to plasmids of *Psychrobacter spp.*

Here we report the characterization of additional 5 isolates in our extended analyses of milk samples (Suppl. Table 1). The BMMF1 isolates described in this study are C1MI.15M.1 (2040nt) (with subtype C1MI.15M.2, 2041nt, 99,9% nucleotide identity, one additional 57aa putative ORF), C1MI.9M.1 (1935nt) (with subtype C1MI.9M.2, 1934nt, 99,7% nucleotide identity, missing 51aa putative ORF), as well as C1MI.3M.1 (1767nt), were all obtained after DNA amplification using back-to-back primers located upstream of the Rep gene. C1MI.3M.1 shares 90% nucleotide identity to the uncultured bacterium plasmid HD4bpcirc (additional accession numbers in Suppl. Table 2) and 83% to our H1MSB.2 isolate by BLASTN analyses. C1MI.15M shares 99% nucleotide identity to uncultured prokaryote plasmid pRGRH0677 from rat gut metagenome, 80% to H1MSB.2 and 78% to pHD4bpcirc, whereas C1MI.9M shares 93% nucleotide identity to the same plasmid pRGRH0677 and 79% to both H1MSB.2 and pHD4bpcirc. These isolates share lower nucleotide identity to the majority of the previous group. Similar to other members of the BMMF1 group, they contain conserved direct repeats (iterons) (4x22 nucleotides, ATAAGACGACACTTACCTACCA) located at nt164-251 in C1MI.15M and at nt162-249 in C1MI.9M. Additional conserved repeats (4x9nt GGTTTTTAA) are located at nt87-121 in C1MI.9M. Similarly, iterons are located at nt164-250 (4x22 ATATCACACCGTTTACCCATCA) in C1MI.3M.

The putative Rep proteins of C1MI.15M and pRGRH0677 are identical (100%), whereas C1MI.9M shares 94% similarity to the rat gut plasmid (Figure 1). The putative Rep protein of C1MI.3M.1 is 95% similar to the Rep protein of pHD4bpcirc, 85% to H1MSB.2Rep and 81% to pRGRH0677Rep. *In silico* analyses of this putative Rep protein point to RepA, as well as to RepC function, differing here from other BMMF1 Rep proteins. In contrast, the putative Rep proteins of the previous group of BMMF1 isolates are only between 55-56% similar to these above mentioned isolates.

The putative 225aa protein from the second large ORF in the C1HB.4 genome (BMMF1) shared 66% identity in 227aa to a mobilization protein (DOUHC7_ACIBA 381aa) identified in Acinetobacter. This protein could be involved in plasmid recombination [28]. It similarly shared 91% identity in 225aa to an uncharacterized protein (N9GYE3_AIHA uncharacterized, 377aa).

The single-stranded *Geminiviridae* (*Begomovirus* and *Mastrevirus* genera) and *Nanoviridae* of plants are associated with small circular ssDNA components which have a nonanucleotide stem-loop origin of replication and encode a Rep protein. They range from 970nt (nanoviruses) to ca 1400 nucleotides (geminiviruses) in size and are dependent on a helper virus for their spread. These alphasatellites have recently been classified into a new family of viruses *Alphasatellitidae* [29]. The functions of these alphasatellites are not clear, although it has been suggested that they contribute to symptoms by influencing transcription rates. The genomes of TT viruses (family *Anelloviridae*) undergo rearrangement during propagation to lead to the formation of small replication competent circular DNA genomes (micro-TTV, ca 700-900nt) [6]. These micro-TTVs encode in part new putative proteins with similarity to cellular proteins suggesting a role in molecular mimicry [6]. The identification of the novel BMMF1 isolates reported in this study also led to the identification of a small circular DNA molecule C1MI.3Ms of 461nt in size. These small circular genomes resulted after PCR amplification using back-to-back primers as described for the full-length genomes. This genome shares 89% nucleotide identity with the plasmid pRGRH0677 isolated from rat gut and 78% and 77% nucleotide identity to H1MSB2 and pHD4bpcirc, respectively. The iteron repeats (4x22nt, ATAAGACGAGACTTACCTACCA) present are identical to that in the genomes of C1MI.15M and C1MI.9M. *In silico* analyses revealed a putative Rep protein of 75 amino acids sharing >95% amino acid similarity to putative Rep proteins of C2MI.15M and C1MI.9M. BLASTP analyses indicate a 97% similarity to the first 50 amino acids of a protein in the Tunicate Oikopleura dioica [30] and lower similarity to uncharacterized RepB proteins.

***In silico characterization of BMMF2 genomes and putative proteins:***

The circular DNA genomes (HCBI1, HCBI2 and HCBI7, Suppl Table 1) were isolated from serum of healthy cattle [17]. These isolates were grouped as BMMF2 based on their nucleotide identity to the previously described Sphinx2.36 [14,31]. In this study we used two sets of back-to-back primers to generate the full-length circular genomes of 97 isolates (Table 1 including accession numbers) ranging from 2102 to 3090 nucleotides. BLASTN comparisons to available DNA databanks, revealed similarity mainly to bacterial plasmids (*Acinetobacter baumannii* str. AYE plasmid p4ABAYE [31], *Acinetobacter baumannii* strain A85 plasmid pA85-1 [32], and *Acinetobacter baumannii* strain DS002 plasmid pTS236 [33], but also to ssDNA isolates from Rat gut (pRGRH0103 and pRGRH0636) [34] (Suppl. Table 2). We performed phylogenetic analyses in order to determine relationships between isolates and to facilitate our selection of isolates with which to continue in-depth biological studies.

We included only one representative of a group of isolates sharing 90-99% nucleotide identity in the phylogenetic tree shown in Fig 2, as well as isolates originating from different dairy samples. Two main clades of were seen – one clade sharing 50% to 57% nucleotide identity to the *Acinetobacter baumanii* plasmid p4ABAYE (Fig.2 clade A) and between 40% and 50% to the second clade (Fig. 2 clade B). The nucleotide identity between pRGRH0636 and pRGRH0103, two circular ssDNA genomes identified by metagenomic analyses from Rat gut, is 39%. The latter shares between 80 to 92% nucleotide identity to C2HB1, C2MI.15B.2, C2MI.16B.3, C2MI.7.16 and C2MI.9B.5

HCBI2.170 was isolated from bovine serum by density gradient fractionation followed by RCA amplification of restricted DNA fragments. Despite using a series of back-to-back primers on the known sequence [17], we failed to rescue a larger fragment. An isolate obtained in the present study resulted in the full-length genome, i.e. HCBI2.170 and C2MI.5A.1 (2257nt) shared identical nucleotides in the overlapping part of the genomes. C2MI.5A.1 now replaces HCBI2.170.

*In silico* analyses of putative open reading frames (ORF, >50aa) were performed on each genome by using the following alternative start codons: ATG, CTG, ATA, ATT, ACG, GTG, TTG and ATC [36]. This resulted in the identification of mainly 2 additional major ORFs on the positive DNA strand (Table 1), as well as putative proteins ranging from 101 to 138 amino acids and 96 to 148 amino acids in size, respectively. Additional smaller ORFs were present. Multiple ORFs (average number 2 to 10, up to 125 amino acids) were identified on the reverse strand. BLASTP analyses of the latter putative proteins revealed no or very weak similarity to known proteins. Further experimental investigation is needed to determine whether splicing events may result in combined, functional proteins. Transcription of the negative DNA strand was previously noted for the BMMF1 group of isolates [21].

An interesting result was the isolation of smaller circular DNA molecules 697nt in size and sharing 93-99% nucleotide identity. These were isolated from 3 different milk samples and one yoghurt sample. *In silico* analyses of C2MIs.5A.1, C2MIs.7A.1, C2MIs.9A.1, and C2MIs.10A.1 revealed 2 overlapping putative ORFs on the positive strand which could encode for 224 and 248 amino acid replication proteins, whereas the negative strand could encode for putative proteins of 53, 58 and 81 amino acids in C2MIs.5A.1 and C2MIs.7A.1, and 64 amino acids in C2MIs.9A.1 and C2MIs.10A.1. The nucleotide sequences of C2MIs.5A.1 and C2MIs.7A.1 were identical to the overlapping regions of the full-length genomes C2MI5B.10 and C2MI.7B.17, respectively. The rep proteins differed from those of the full-length genomes counterparts, mainly in N-terminal 33aa of 248aa rep proteins and 7aa of both 224aa and 248aa proteins in the C-terminal. In addition, single amino acid differences were noted between the 2 groups of small circular genomes.

As the aim of our study is to identify, isolate and associate unknown infectious agents to the etiology of colon cancer, we performed mass spec analyses of protein samples from colon cancer and normal tissue, as well as laser microdissection of these samples. Mass spec analyses of protein samples from colon cancer and normal colon tissue (Bund et al., in preparation), resulted in identification of multiple specific peptide motifs originating from 3 BMMF2 isolates. Specific peptide motifs could be allocated, 5 each to C2MI.15B.16 and C2MI.9B.10 (Table 2). Interestingly, several of these amino acid motifs indicating a possible functional involvement, were located on the negative DNA strand. Isolates C2MI.10A.1 and C2MI.10A.2 share 99.7% nucleotide identity, but 3 specific peptide motifs were identified in mass spec to C2MI.10A.1, whereas C2MI.10A.2 harbored only 2 of these 3 motifs. BMMF isolates sharing >90% identity in their nucleotide sequences, were initially regarded as variants of a single genome. Based on the possible biological significance of such small differences, we decided to include 97 BMMF2 isolates for more detailed analyses. In addition, we recovered a member of the BMMF1 group from colon tissue by laser microdissection. Cloning and sequencing confirmed a H1MSB.1 genome sequence modified from the original isolate MSB1.176 (now H1MSB.1) from an autopsy brain sample of a multiple sclerosis patient (Fig 4a). The nucleotide modifications included mainly C/T or T/C or less often, A/G or G/A transitions. These led to alterations in the putative gene organization with an additional 62aa putative protein in LD10.154 (Fig 4b). Domainsweep analyses indicated a weak similarity to a domain (diarrhea inducing) of a non-structural glycoprotein of Rotaviruses.

***Characteristics specific for putative BMMF replication proteins:***

Bacterial plasmids are classified based on nucleotide homology of their replicase genes [37]. We identified 2 overlapping putative Rep genes in the BMMF2 isolates differing between 30aa to 50aa in length. Putative Rep proteins of all BMMF2, as well as BMMF1 isolates were compared. A core conserved region (329aa) was used in the phylogenetic analyses. BMMF2 rep proteins were grouped into 2 clades (A and B), whereas BMMF1 rep proteins formed a third clade (C) (Fig.1). Clades A and B shared 60 to 65% similarity. Rep proteins in clade A shared 69-90% similarity within the clade and 70-82% to the p4ABAYE rep. Similarly clade B rep proteins shared 80-90% within the clade and 59-62% to p4ABAYE rep. The rep proteins of BMMF1 (clade C) are more diverse and shared almost no similarity (10-12%) to clades A and B. The rep proteins of H1MSB.1, C1MI5.1, and C1MI.5.2 are identical and share 97% identity to that of Sphinx1.76 [31]. These all share 75-88% similarity to C1MI.1, C1MI.2, C1MI.3, C1MI.4 and C1HB.6.2 reps. This group of rep proteins share only 45% identity to C1HB.4rep, 37 to 39% to C1HB.5rep and 58% to H1MSB.2rep. The Rep proteins of the newly identified BMMF1 isolates described in this study, share 50-62% to the other BMMF1 isolates.

*In silico* structure prediction [38] of putative BMMF Reps revealed a partial structural homology of Rep with known replication initiator proteins of the Rep superfamilies 1,2 and 3. Catalytically active motifs previously identified in the Reps of Geminivirus and other known ssDNA viruses were identified in the BMMF2 Reps [3, 39,40]. Motif I (consensus aa FLTYP) is generally less conserved and also partially represented in BMMF2 Reps (T(F/L)(T/S)V(K/R)N). Motif II (consensus HxH) is important for metal ion binding and is – with one exception C2MI.5B.10rep – present as H(V/L/I)H in all BMMF2 reps. Motif III containing the active site tyrosine is present as consensus YALK in all BMMF2 reps. Motif positions in the C2HBI7Rep, taken as representative, are as follows: motif I at aa 110-115, motif II at 172-174 and motif III at aa 227-230. The lengths of amino acid spacers between the motifs in C2HBI7Rep are roughly 60aa between motifs I and II and 50aa between motif II and III. Putative Walker A, B and C (SF3 helicase) motifs are not conserved amongst the BMMF2 Reps. Structure predictions based on amino acid sequences of BMMF1 Reps show similarity to RepB within the Rep superfamily 3 which also harbors RepE, RepC and RepA. Crystallography based on the N-terminal domain of the H1MSB.1 Rep (aa1-136) resulted in a structure with a resolution of 1.53 [41]. The overall structure showed a remarkable similarity with the structure of the RepA protein of *Pseudomonas syringae* despite sharing only 28% amino acid sequence identity.

The putative rep proteins of 14 BMMF2 isolates did not result only from one continuous ORF, but were compiled of more than one truncated ORF. BLASTP analyses of the smaller putative proteins indicated similarity to the Rep gene. A similar phenomenon was observed in a previous study on TT viruses (*Anelloviridae*). Here the premature stop codons had been verified and resulted from confirmed single nucleotide differences between independent isolates of the same TTV type [16]. In order to ascertain whether potential introns were present in BMMF2 genomes (with and without continuous Rep genes), we performed GENSCAN analyses [23]. This prediction tool was developed to identify genes in prokaryotes, eukaryotes and plants and was most suitable for our present purpose. We aligned the resulting genes to the respective gene maps obtained on the full-length nucleotide genomes. The GENSCAN result of *Acinetobacter baumanii* plasmid p4ABAYE is included (Fig. 3, Suppl. Table 3). The closest related isolate with an intact Rep ORF is presented with each isolate with truncated Rep ORFs. The presence in almost every isolate of a polyadenylation (poly(A) signal (restricted to 6nt) is very notable in the GENSCAN products, including in the plasmid p4ABAYE. The polyA signal (AATAAA) of the latter was identical to the majority of putative poly(A) signals in the BMMF2 group. Variations (ATAAAA, AAATAA) were detected in 9 other isolates. Notable differences were AATCAA in C2MI.5A.2 and CTTAAA in C2MI.16B.2.

No significant hits were obtained in the majority of BLASTP analyses performed on most of the additional putative proteins identified in various BMMF2 isolates. DomainSweep analyses provide a compilation of protein characteristics resulting from identification of protein structures by employing different protein database search methods and scanning a number of protein domain/family databases [22, 42]. The minority of additional putative proteins identified in individual isolates shared very weak similarities (i.e. not significant similarities, but nevertheless included in the first 10 listed for the respective search method used in Domainsweep) to proteins with known functions. In view of the aim of the present study, we regard these data as indicative of possible functions, but needs to be verified by further investigation. Interestingly a few putative Rep proteins were assigned very weak similarities to RNA helicase, to the RNA-binding S4 domain and to RNA polymerase II Rpb4 core domain, as well as a putative protein from region 5 (116aa) to a specific subgroup of CDD/CDA-like deaminases [43] (Table 1). The H1MSB.1 isolate recovered from colon tissue displayed mainly C/T modifications. These transitions led to alterations in the genome structure (Fig 4) resulting in putative genes which could be involved in pathogenesis. Small ssDNA viruses experience higher mutation rates when in a changing environment such as chronic infections or host immune defence [44]. These modifications could also lead to more virulent viral strains inducing severe disease [45]. RNA editing of hepatitis D virus occurs via C/T or on the negative strand as a functional A-to-G substitution [46]. The unexpected similarities to RNA helicase and deaminase, the perfect matching peptides (identified in mass spec) which are allocated to the negative strand of the respective genome, as well as the two overlapping putative Rep genes, posed the question to the origin of these BMMF2 isolates which were initially all obtained by DNA amplification. These characteristics are very conspicuous and stimulated speculation as to whether parallels can be drawn to the structure and replication of Hepatitis delta virus [47], but this requires further investigation.

In addition, the following two results deserve discussion: C2HB7 (BMMF2) codes for a putative 228aa protein sharing a weak similarity to the bacterial ribbon-helix-helix CopG protein which acts as a transcriptional repressor on the Rep gene [48]. The putative 291aa protein of C1MI.1 (BMMF1) also shares weak similarity to the CopG protein. Surprisingly this 228aa putative protein of C2HB7 displays similarity to viral proteins as well, i.e. it shares weak homology to the capsid protein of narcissus mosaic RNA virus reflecting recombination between DNA and RNA viruses during evolution [49]. Bacilladnaviruses (with a partial double-stranded DNA genome) probably acquired their capsid gene from ssRNA nodaviruses by horizontal transfer [11].

Possible recombination events within the BMMF2 group were also noted. The 196aa putative protein of C2HB7 shares 90% identity with the 183aa putative protein of C2MI.3A.2, whereas their rep proteins share only 45-55% identity (conserved core 64%). The nucleotide identity between these full-length genomes is 55%. Possible recombination events were noted in aligning the full-length nucleotide genomes of all BMMF2 isolates. This observation needs to be confirmed by additional phylogenetic analyses which were not covered in this study.

**Discussion:**

Infectious agents are involved in the pathogenesis of a number of malignant diseases [8]. Based on available epidemiological data, we intensified our search for yet unknown infectious agents in additional cancer types [8,14]. We described the isolation of BMMF factors from serum obtained from healthy cattle, as well as brain and serum from patients suffering multiple sclerosis [14]. Closer characterization of these isolates not only revealed similarities to plasmids from *Acinetobacter baumanii*, but included features of circular ssDNA viruses in plants and animals. The recovery of a BMMF1 isolate H1MSB1 from the peritumoral lamina propria cells of colon cancer patients with CD68 macrophage invasion [15] also demonstrates the association of these agents to infections of the colon. These results potentially place such infectious agents into a new class of pathogens [15].

*Acinetobacter baumanii* is broadly distributed in the environment and commonly found in water, soil and animals [50]. Classification of the *Acinetobacater baumanii* plasmids is based on typing of the replicons and replicase genes [37]. These plasmids typically have 4 conserved direct repeats (iterons) and a Rep gene belonging to the Rep_3 superfamily or no iterons when comprising a Rep gene belonging to the Rep1/2 superfamily. Isolates of the BMMF1 group were related to a broader range of bacterial plasmids. They harbor iterons and have genes encoding initiator of replication proteins (RepB or Rep superfamily 3). RepB does not have an intrinsic helicase activity, but retains this activity through formation of a hexameric ring structure [51]. The latter is also a necessity for the helicase activity in eukaryotic viruses. One putative protein of isolate C1HB4 has weak similarity to a mobilization protein. In contrast, the closest related sequence to the BMMF2 isolates is plasmid p4ABAYE, the smallest of 4 plasmids of *Acinetobacter baumanii* strain ABAYE. This plasmid has been described as cryptic and is classified together with plasmids pTS236 and pA85-1 in the GR14 clade [33,37,52]. Plasmid p4ABAYE is unique and seems to have followed a different evolutionary pathway than other bacterial plasmids. Distinct differences are that p4ABAYE has no iterons, has lost its mobility, with only the replication (Rep_1 gene) and transfer functions remaining, and did not share any link with the 6 chromosomes of *Acinetobacter baumanii*, i.e. does not participate in exchange of genes [50]. The BMMF2 isolates share characteristics with plasmid p4ABAYE, i.e. size of genome (2726nt), nucleotide similarity, do not have iterons and have a gene encoding a putative Rep protein belonging to the Rep superfamily 1/2.

BMMF1 and BMMF2 also display viral features. The Rep genes of recently identified CRESS (circular Rep-encoding ssDNA)-DNA viruses are highly conserved and form a hexameric ring structure [3]. These ssDNA viruses (which include the plantviruses *Geminiviridae* and *Nanoviridae)* are all characterized as having a stem-loop structure as the origin of replication. Such a putative stem-loop structure is present in BMMF1 isolates (in addition to the bacterial iterons). The second large ORF of several CRESS-DNA viruses shows some similarity to capsid proteins [3] – a similar observation was made for single BMMF2 isolates. The *in silico* observation of “truncated” ORFs which putatively code for a Rep protein may represent a similar mechanism of transcription as described in plant RNA viruses. Here the non-canonical translation includes leaky scanning, stop-codon read-through and ribosomal frameshifting [53].The presence of polyA signal in almost all putative transcripts including that of p4ABAYE, also indicate transcription in eukaryotes.

The most striking feature of both BMMF1 and BMMF2 groups is the existence of larger full-length genomes in conjunction with smaller circular satellite-like genomes encoding for only Rep-associated genes. The bacterial iterons are also present in smaller genomes in BMMF1. Members of the recently established family *Alphasatellitidae* all have a stem-loop structure as origin of replication. Although their specific function remains unclear, their co-infection is associated with exacerbation or reduction of disease symptoms in plants [29]. Similarly, the non-coding deltasatellites (682nt) associated with genus Begomovirus of the *Geminiviridae* seem to influence the severity of disease or virus accumulation in plants indirectly [54]. Co-infection of a “large” (C1MI.1, 2523nt) and “small” (H1MB1, 1776nt) BMMF1 isolates resulted in increased transcription of the smaller genome [20]. The identification of even smaller circular genomes in both BMMF1 and BMMF2 - C1MI.3Ms (461nt with a 75aa putative Rep protein), C2MIs.5A.1, C2MIs.7A.1, C2MIs.9A.1, and C2MIs.10A.1 (697nt encoding for a putative full-length Rep protein), is intriguing. Future *in vivo* investigations are urgently needed exploring their functions, as this may indicate whether these molecules act like viral satellites influencing or even causing disease symptoms.

This group of infectious agents has been implicated in the pathogenesis of colon and breast cancer [15]. Although the evolutionary origin is unknown, it seems to fall between a unique bacterial plasmid and circular ssDNA viruses. We suspect that it originated from a bacterial plasmid which has evolved to adapt to infect and replicate in mammalian cells. The acquisition of smaller satellite-like genomes may have evolved subsequently in order to strengthen and/or extend survival or to convey pathogenic symptoms. Indirect hints as to the origin and structure of our isolates, as well as its replicative behavior, result from parallels drawn from Hepatitis deltavirus genome structure and replication.

Thus far, the present available analyses of human whole genome sequencing precluded the identification of originally bacterially-derived plasmids as human pathogens by dismissing and discarding sequences suspected to originate from bacterial contaminants. A different perspective for investigating an unexplored spectrum of potential human pathogens seems to be highly desirable and recommended.

**Acknowledgements:** We thank Imke Grewe for excellent technical assistance. This study was funded by an unrestricted grant from Oryx, München.

**Declaration of Interests:**

The authors declare no competing interests.

**References:**

1. Kowarsky M, Camunas-Soler J, Kertesz M, et al. Numerous uncharacterized and highly divergent microbes which colonize humans are revealed by circulating cell-free DNA. [Proc Natl Acad Sci U S A.](https://www.ncbi.nlm.nih.gov/pubmed/?term=Kowarsky%2C+M.%2C+Camunas-Soler%2C+J.%2C+Kertesz%2C+M.%2C+De++Vlaminck%2C+I.%2C+Koh%2C+W.%2C+Pan%2C+W.%2C+Martin%2C+L.%2C+Neff%2C+N.F.%2C+Okamoto%2C+J.%2C+Wong%2C+R.J.%2C+et+al.+(2017).) 2017; 114:9623-9628.

2. Fernandes MA, Verstraete SG, Phan TG, et al. (2019) Enteric virome and bacterial microbiota in children with ulcerative colitis and Crohn's disease. J Pediatr Gastroenterol Nutr 2019; 68:30-36.

3. Zhao L, Rosario K, Breitbart M, et al. Eukaryotic circular Rep-encoding single-stranded DNA (CRESS DNA) viruses: Ubiquitous viruses with small genomes and a diverse host range. [Adv Virus Res.](https://www.ncbi.nlm.nih.gov/pubmed/?term=zhao+rosario+breitbart) 2019; 103:71-133.

4. Gronenborn B, Randles JW, Knierim D, et al. Analysis of DNAs associated with coconut foliar decay disease implicates a unique single-stranded DNA virus representing a new taxon. Sci Rep 2018; 8:5698.

5. Wang H, Li S, Mahmood A, et al. Plasma virome of cattle from forest region revealed diverse small circular ssDNA viral genomes. Virol J. 2018; 15:11.

6. de Villiers EM, Borkosky SS, Kimmel R, et al. The diversity of torque teno viruses: in vitro replication leads to the formation of additional replication-competent subviral molecules. J Virol. 2011; 85:7284-7295.

7. Peretti A, FitzGerald PC, Bliskovsky V, et al. Hamburger polyomaviruses. J Gen Virol. 2015; 96:833–839.

8. zur Hausen H, de Villiers EM. Dairy cattle serum and milk factors contributing to the risk of colon and breast cancers. Int J Cancer 2015; 137:959-967.

9. Moustafa A, Xie C, Kirkness E, et al. The blood DNA virome in 8,000 humans. PLoS Pathog. 2017; 13:e1006292.

10. Sadeghi M, Kaupusinsky B, Yugo DM, et al. Virome of US bovine calf serum. Biologicals 2017; 46:64-67.

11. Kazlauskas D, Dayaram A, Kraberger S, et al. Evolutionary history of ssDNA bacilladnaviruses features horizontal acquisition of the capsid gene from ssRNA nodaviruses. Virology 2017; 504:114-121.

12. Krupovic M, Zhi N, Li J, et al. Multiple layers of chimerism in a single-stranded DNA discovered by deep sequencing. Genome Biol Evol. 2015; 7:993-1001.

13. Roux S, Enault F, Bronner G, et al. Chimeric viruses blur the borders between the major groups of eukaryotic single-stranded DNA viruses. Nat Commun. 2013; 4:2700.

14. zur Hausen H, Bund T, de Villiers EM. Infectious agents in bovine red meat and milk and their potential role in cancer and other chronic diseases. Curr Top Microbiol Immunol. 2017; 407:83-116.

15. zur Hausen H, Bund T, de Villiers EM. Specific nutritional infections early in life as risk factors for human colon and breast cancers several decades later. Int J Cancer 2019; 144:1574-1583.

16. Jelcic I, Hotz-Wagenblatt A, Hunziker A, et al. Isolation of multiple TT virus genotypes from spleen biopsy tissue from a Hodgkin's disease patient: genome reorganization and diversity in the hypervariable region. J Virol. 2004; 78:7498-7507.

17. Funk M, Gunst K, Lucansky V, et al. Isolation of protein-associated circular DNA from healthy cattle serum. Genome Announc 2014; 2:e00846-14.

18. Gunst, K, zur Hausen H, de Villiers EM. Isolation of bacterial plasmid-related replication-associated circular DNA from a serum sample of a multiple sclerosis patient. Genome Announc. 2014; 2(4). pii: e00847-14.

19. Whitley C, Gunst K, Müller H, et al. Novel replication-competent circular DNA molecules from healthy cattle serum and milk and multiple sclerosis-affected human brain tissue. Genome Announc. 2014; 2. pii: e00849-14.

20. Lamberto I, Gunst K, Müller H, et al. Mycovirus-like DNA virus sequences from cattle serum and human brain and serum samples from multiple sclerosis patients. *Genome Announc.* 2014; 2, pii: e00848-14.

21. Eilebrecht S, Hotz-Wagenblatt A, Sarachaga V, et al. Expression and replication of virus-like circular DNA in human cells. Sci Rep 2018; 8:2851.

38. del Val C, Ernst P, Falkenhahn M, et al. ProtSweep, 2Dsweep and DomainSweep: protein analysis suite at DKFZ. Nucl Acids Res 2007; W444-450.

23. Burge C, Karlin S. Prediction of complete gene structures in human genomic DNA. J Mol Biol 1997, 268:78-94.

24. Stanke M, Morgenstern B. AUGUSTUS: a web server for gene prediction in eukaryotes that allows user-defined constraints. Nucl Acid Res 2005; 33:W465-467.

25. Besemer J, Borodovsky M. GeneMark: web software for gene finding in prokaryotes, eukaryotes and viruses. Nucl Acid Res 2005; 33:W451-454

26. Kumar S., Stecher G., Li M., Knyaz C., and Tamura K. MEGA X: Molecular Evolutionary Genetics Analysis across computing platforms. Molecular Biology and Evolution 2018; 35:1547-1549.

27. Falida K, Eilebrecht S, Gunst K, et al. Isolation of two virus-like circular DNAs from commercially available milk samples. Genome Announc. 2017; 5.pii: e00266-17.

28. Priebe SD, Lacks SA. Region of the Streptococcal plasmid pMV158 required for conjugative mobilization. J Baterio.l 1989, 171:4778-4784.

29. Briddon RW, Martin DP, Roumagnac P, et al. Alphasatellitidae: a new family with two subfamilies for the classification of geminivirus- and nanovirus-associated alphasatelllites. Arch Virol. 2018; doi.org/10.1007/s00705-018-3854-2.

30. Denoeud F, Henriet S, Mungpakdee S, et al. Plasticity of animal genome architecture unmasked by rapid evolution of a pelagic tunicate. Science 2010; 330:1381-1385.

31. Manuelidis L. Nuclease resistant circular DNAs copurify with infectivity in scrapie and CJD. J Neurovirol. 2011; 17:131-145.

32. Vallenet D, Nordmann P, Barbe V, et al. Comparative analysis of Acinetobacters: three genomes for three lifestyles PLoS ONE 2008, 3: E1805.

33. Hamidian M, Kenyon JJ, Holt KE, et al. A conjugative plasmid carrying the carbapenem resistance gene blaOXA-23 in AbaR4 in an extensively resistant GC1 Acinetobacter baumannii isolate. J Antimicrob Chemother. 2014; 69:2625-2628.

34. Longkumer, T., Kamireddy, S., Muthyala, V.R., et al. Acinetobacter phage genome is similar to Sphinx 2.36, the circular DNA copurified with TSE infected particles. Sci Rep. 2013; 3:2240.

35. Jorgensen TS, Hansen MA, Xu Z, et al. Plasmids, viruses, and other circular elements in rat gut. 2017; <https://www.biorxiv.org/content/10.1101/143420v2.abstract>

36. Kearse MG, Wilusz JE. Non-AUG translation: a new start for protein synthesis in eukaryotes. Genes Develop. 2017; 31:1717-1731.

37. Bertini A, Poirel L, Mugnier PD, et al. Characterization and PCR-based replicon typing of resistance plasmids in Acinetobacter baumannii. Antimicrob Agents Chemother. 2010; 54:4168-4177.

38. Kelley LA, Mezulis S, Yates CM, et al. The Phyre2 web portal for protein modeling, prediction and analysis. Nat Protoc 2015; 10:845-858.

39. Campos-Olivas R, Louis JM, Clerot D, et al. The structure of a replication initiator unites diverse aspects of nucleic acid metabolism. Proc Natl Acad Sci USA. 2002; 99:10310-10315.

40. Varsani A, Krupovic M. Sequence-based taxonomic framework for the classification of uncultured single-stranded DNA viruses of the family *Genomoviridae*. Virus Evol. 2017; 3:vew037.

41. [Kilic T](https://www.ncbi.nlm.nih.gov/pubmed/?term=Kilic%20T%5BAuthor%5D&cauthor=true&cauthor_uid=31063152), [Popov AN](https://www.ncbi.nlm.nih.gov/pubmed/?term=Popov%20AN%5BAuthor%5D&cauthor=true&cauthor_uid=31063152), [Burk-Körner A](https://www.ncbi.nlm.nih.gov/pubmed/?term=Burk-K%C3%B6rner%20A%5BAuthor%5D&cauthor=true&cauthor_uid=31063152), [Koromyslova A](https://www.ncbi.nlm.nih.gov/pubmed/?term=Koromyslova%20A%5BAuthor%5D&cauthor=true&cauthor_uid=31063152), [Zur Hausen H](https://www.ncbi.nlm.nih.gov/pubmed/?term=Zur%20Hausen%20H%5BAuthor%5D&cauthor=true&cauthor_uid=31063152), [Bund T](https://www.ncbi.nlm.nih.gov/pubmed/?term=Bund%20T%5BAuthor%5D&cauthor=true&cauthor_uid=31063152), [Hansman GS](https://www.ncbi.nlm.nih.gov/pubmed/?term=Hansman%20GS%5BAuthor%5D&cauthor=true&cauthor_uid=31063152). Structural analysis of a replication protein encoded by a plasmid isolated from a multiple sclerosis patient. [Acta Crystallogr D Struct Biol.](https://www.ncbi.nlm.nih.gov/pubmed/?term=rep+protein+structure+bund-t) 2019; 75:498-504. doi: 10.1107/S2059798319003991.

42. Senger, M., Flores, T., Glatting, K.-H., et al. W2H: WWW interface to the GCG sequence analysis package. Bioinformatics 1998; 14:452-457. http://www.w2h.dkfz-heidelberg.de.

43. Iyer LM, Zhang D, Rogozin IB, et al. Evolution of the deaminase fold and multiple origins of eukaryotic editing and mutagenic nucleic acid deaminases from bacterial toxin systems. Nucl Acids Res. 2011; 39:9473-9497.

44. Duffy S, Shackleton LA, Holmes EC. Rates of evolutionary change in viruses: patterns and determinants. Nature Rev. Genetics 2008; 9:267-276.

45. Firth C, Charleston MA, Duffy S, Shapiro B, Holmes EC. Insights into the evolutionary history of an emerging livestock pathogen: Porcine circovirus 2. J Virol 2009; 83: 12813-12821.

46. Casey JL, Gerin JL. Hepatits D virus RNA editing: Specific modification of adenosine in the antigenomic RNA. J Virol. 1995; 69:7593-7600.

47. Sureau C, Negro F. The hepatitis delta virus: Replication and pathogenesis. J Hepatol. 2016; 64:S102-S116.

48. Gomis-Rüth FX, Solá M, Acebo P, et al. The structure of plasmid-encoded transcriptional repressor CopG unliganded and bound to its operator. EMBO J 1998; 17:7404-7415.

49. Stedman KM. Deep recombination: RNA and ssDNA virus genes in DNA virus and host genomes. Annu Rev Virol. 2015; 2:203-217.

50. Fondi M, Bacci G, Brilli M, et al. Exploring the evolutionary dynamics of plasmids: the Acinetobacter pan-plasmidome. BMC Evol Biol. 2010; 10:59.

51. Boer DR, Ruiz-Masó JA, Rueda M, et al. Conformational plasticity of RepB, the replication initiator protein of promiscuous streptococcal plasmid pMV158. Sci Rep. 2016; 6:20915.

52. Lean SS, Yeo CC. Small, enigmatic plasmids of the nosocomial pathogen, Acinetobacter baumannii: Good, Bad, Who Knows? Front Microbiol. 2017; 8:1547.

53. Miras M, Miller WA, Truniger V, et al. Non-canonical translation in plant RNA viruses. Front Plant Sci. 2017; 8:494.

54. Hassan I, Orillio AF, Fiallo-Olivé E, et al. Infectivity, effects on helper viruses and whitefly transmission of the deltasatellites associated with sweepoviruses (genus Begomovirus, family Geminiviridae). Sci Rep 2016; 6: 30204.

55. Jones DT, Taylor WR, Thornton JM. The rapid generation of mutation data matrices from protein sequences. Comp Appl Biosci 1992, 8:275-282.

56. Felsenstein J. Confidence limits on phylogenies: An approach using the bootstrap. Evolution 1985; 39:783-791.

57. Tamura K. and Nei M. Estimation of the number of nucleotide substitutions in the control region of mitochondrial DNA in humans and chimpanzees. Molecular Biology and Evolution 1993; 10:512-526.

***Legends to the figures:***

**Figure 1**: Molecular phylogenetic analysis of the overlapping region of all Rep proteins of both BMMF1 and BMMF2.

The evolutionary history was inferred by using the Maximum Likelihood method and JTT matrix-based model [55]. The bootstrap consensus tree inferred from 500 replicates [56] is taken to represent the evolutionary history of the taxa analyzed [56]. Branches corresponding to partitions reproduced in less than 50% bootstrap replicates are collapsed. The percentage of replicate trees in which the associated taxa clustered together in the bootstrap test (500 replicates) are shown next to the branches [56]. Initial tree(s) for the heuristic search were obtained by applying the Neighbor-Joining method to a matrix of pairwise distances estimated using a JTT model. This analysis involved 104 amino acid sequences. There were a total of 319 positions in the final dataset. Evolutionary analyses were conducted in MEGA X [26].

**Figure 2:** Phylogenetic tree of the nucleotide sequences of representatives of each BMMF2 isolate in comparison to the plasmids of *Acinetobacter baumanii* p4ABAYE, pA85-1 and pTS236, genomic isolates from rat gut pRGRH0103 (RH103) and pRGRH0636 (RH0636) in clade A, as well as Sphinx2.36 and our previous BMMF1 isolates C2MI1 and C2HB7 in clade B. The recently isolated C2MI.5A.1 represents the full-length genome of C2HB2. The stars indicate BMMF2 isolates of which the nucleotide sequence is identical in the overlapping nucleotide sequence of the respective “small” BMMF2 isolate – C2MI.7B.17 to C2MIs.7A.1 and C2MI.5B.10 to C2MIs.5A.1. The evolutionary history was inferred by using the Maximum Likelihood method and Tamura-Nei model [57]. The bootstrap consensus tree inferred from 500 replicates [56] is taken to represent the evolutionary history of the taxa analyzed [56]. Branches corresponding to partitions reproduced in less than 50% bootstrap replicates are collapsed. The percentage of replicate trees in which the associated taxa clustered together in the bootstrap test (500 replicates) are shown next to the branches [56]. Initial tree(s) for the heuristic search were obtained by applying the Neighbor-Joining method to a matrix of pairwise distances estimated using the Maximum Composite Likelihood (MCL) approach. This analysis involved 48 nucleotide sequences. There were a total of 3170 positions in the final dataset. Evolutionary analyses were conducted in MEGA X [26].

**Figure 3:** Schematic presentation of the ORF maps of BMMF2 isolates displaying truncated ORFs which encode for putative Rep proteins (arrows in red) in comparison to closely related isolates with intact Rep ORFs (blue arrows). The ORF map of p4ABAYE is shown in the middle. Arrows in same color (green or yellow) represent ORFs which encode putative proteins showing high similarity between isolates. The GENESCAN analyses indicating the resulting splicing transcriptional events are indicated in purple. The large purple dot represents the poly A signal. In some cases no poly A signal is present for the respective transcript.

**Figure 4:** Modified DNA genomes of BMMF1 H1MSB.1 were recovered by laser microdissection from the lamina propria cells of the colon from colon cancer patients. A – area dissected from the peritumoral colon tissue. Rep-stained areas are marked (yellow). Bar 100μm. B - Clustal analysis of the modified genomes LD10.154 and LD10.158 with the original H1MSB.1 genome indicated a number of C/T transitions (light grey shaded) and fewer A/G modifications (dark grey shaded). C – Schematic presentation of the putative gene organization of these DNA genomes. The modifications in the LD10.154 genome led to a modified gene structure (red arrow) resulting in a 62 amino acid putative protein which could be involved in pathogenesis.
