# Supplementary material for: A specific class of infectious agents isolated from bovine serum and dairy products and peritumoral colon cancer tissue
Source: Emerg Microbes Infect. 2019 Aug 14;8(1):1205–18. doi: 10.1080/22221751.2019.1651620 (PMC6713099; doi:10.1080/22221751.2019.1651620)
Supplement: Supplemental Material [file TEMI_A_1651620_SM3013.zip › suppl_data/supl_Table_2_proof_3.8.19.docx]

**Supplementary Table 2: Other isolates mentioned in this study**

| **Isolate** | **Genome size (nt)** | **Accession no.** | **Reference** |
| --- | --- | --- | --- |
| HD4bpcirc | 1881 | KX838913.1 | Biagini et al., unpub |
| pRGRH0677 | 2041 | LN853297 | [37] |
| Sphinx1.176 | 1758 | HQ444404 | [33] |
| Sphinx2.36 | 2360 | HQ444405 | [33] |
| *Acinetobacter baumannii* str. AYE plasmid p4ABAYE, | 2726 | CU459139 | [34] |
| *Acinetobacter baumannii* strain A85 plasmid pA85-1, | 2726 | CP021783 | [35] |
| *Acinetobacter baumannii* strain DS002 plasmid pTS236 | 2252 | JN872565 | [36] |
| pRGRH0103 | 2309 | LN852793 | [37] |
| pRGRH0636 | 2579 | LN853262 | [37] |
| Tomato yellow leaf curl virus (TYLCV) |  | CAA43466 | [41] |
