# Supplementary material for: A specific class of infectious agents isolated from bovine serum and dairy products and peritumoral colon cancer tissue
Source: Emerg Microbes Infect. 2019 Aug 14;8(1):1205–18. doi: 10.1080/22221751.2019.1651620 (PMC6713099; doi:10.1080/22221751.2019.1651620)
Supplement: Supplemental Material [file TEMI_A_1651620_SM3013.zip › suppl_data/supl_Table_3_20.7.19_ohne.docx]

| **Supplementary Table 3:** **BMMF2 isolates – putative transcription products indicated by GENSCAN (re: Fig 3)** | | | | | | |
| --- | --- | --- | --- | --- | --- | --- |
| **Isolate** | **Intr** | **Intr** | **Intr** | **Term** | **polyA signal** | **Product (aa)** |
| p4ABAYE | 136-993 |  |  | 1776-2102 | 2319-2324 | 394 |
| C2MI.1A.1 | 54-924 | 1125-1245 | 1537-1830 | 2001-2131 | 2231-2236 | 472 |
| C2MI1A.3 | 438-925 | 1126-1246 | 1538-1831 | 2002-2132 | 2232-2237 | 344 |
| C2MI10A.1 | 88-699 | 738-926 |  | 1476-1904 | 2434-2439 | 409 |
| C2MI10A.2 |  |  |  | 88-948 | 2442-2447 | 286 |
| C2MI15B.14 | 45-1039 | 1865-2131 |  | 2358-2605 | 2705-2710 | 503 |
| C2MI16B.9 | 267-1117 | 1976-2132 |  | 2359-2606 | 2706-2711 | 418 |
| C2MI16B.11 | 267-1117 | 1977.2133 |  | 2400-2611 | 2711-2716 | 406 |
| C2MI5B.6 | 45-1219 | 1552-1722 |  | 1902-2206 | 2305-2310 | 550 |
| C2MI5B.7 | 496-1219 | 1551-1896 |  | 1902-2205 | 2304-2309 | 457 |
| C2MI5B.12 | 226-651 | 717-1115 | 2147-2333 |  | ----- | 325 |
| C2MI9B.4 | 495-1209 |  |  | 1496-1824 | 2207-2212 | 347 |
| C2MI9B.7 | 45-1210 |  |  | 1497-1825 | 2208-2213 | 498 |
| C2MI9B.10 | 266-622 | 738-1129 | 2413-2599 |  | ------ | 313 |
|  |  |  |  |  |  |  |
|  |  |  |  |  |  |  |
| **Isolate** | **Promoter** |  | **Intr** | **Intr** | **polyA signal** | **Product (aa)** |
| C2MI15B.7 |  |  |  | 88-948 | 1226-1231 | 224 |
|  | 1449-1488 |  |  | 1514-1822 | 2208-2213 | 107 |
| C2MI15B.13 |  |  |  | 564-947 | 1225-1230 | 127 |
|  | 1448-1487 |  |  | 1513-1821 | 2207-2212 | 107 |
| C2MI16B.7 |  |  |  | 266-1008 | 1344-1349 | 247 |
|  | 1731-1770 |  |  | 1795-2109 | 2234-2239 | 104 |
| C2MI7A.4 |  |  | 54-474 | 480-956 | 1316-1321 | 299 |
|  | 1493-16582 |  |  | 1858-2151 | 2251-2256 | 97 |
| C2MI7A.5 |  |  |  | 253-957 | 1317-1322 | 234 |
|  | 1494-1533 |  |  | 1859-2152 | 2253-2258 | 97 |
| C2MI7A.6 |  |  |  | 54-957 | 1317-1322 | 301 |
|  | 1494-1533 |  |  | 1859-2152 | 2253-2258 | 97 |
| C2MI7B.15 |  |  |  | 497-1160 | 1328-1333 | 321 |
|  | 1738-1777 |  | 1798-1953 | 2144-2315 | ----- | 110 |
| C2MI7B.17 |  |  |  | 266-1161 | 1329-1334 | 298 |
|  | 1739-1778 |  | 1799-1954 | 2145-2316 | ----- | 110 |
| C2MI7B.18 |  |  |  | 267-1162 | 1330-1353 | 298 |
|  | 1740-1779 |  | 1800-1955 | 2146-2317 | ------ | 110 |
|  |  |  |  |  |  |  |
|  |  |  |  |  |  |  |
| *BMMF1 isolates recovered by laser microdissection from colon tissue* | | | | | | |
| Isolate | **Intr** |  |  | **Term** | **polyA signal** | **Product (aa)** |
| H1MSB.1 | 48-713 |  |  |  | 1234-1239 | 221 |
| LD10.154 | 48-465 |  |  | 871-980 | 1029-1034 | 175 |
| LD10.158 | 48-642 |  |  | 899-981 | 1235-1240 | 225 |
