# Supplementary material for: A specific class of infectious agents isolated from bovine serum and dairy products and peritumoral colon cancer tissue
Source: Emerg Microbes Infect. 2019 Aug 14;8(1):1205–18. doi: 10.1080/22221751.2019.1651620 (PMC6713099; doi:10.1080/22221751.2019.1651620)
Supplement: Supplemental Material [file TEMI_A_1651620_SM3013.zip › suppl_data/suppl_Table_1_6.6.19_ohne.docx]

**Supplementary Table 1: Previously published BMMF1 and BMMF2 isolates, as well as newly identified BMMF1 isolates**

| **BMMF1** | | | | |
| --- | --- | --- | --- | --- |
| **Previous isolates** | | | | |
| **Previous isolates** | **New annotation** | **Genome size (nt)** | **Acc. No.** | **Reference** |
| MSBI1.176 | H1MSB.1 | 1766 | LK931491 | [19] |
| MSBI2.176 | H1MSB.2 | 1766 | LK931492 | [19] |
| CMI1.252 | C1MI.1 | 2523 | LK931487 | [19] |
| CMI2.214 | C1MI.2 | 2148 | LK931488 | [19] |
| CMI3.168 | C1MI.3 | 1687 | LK931489 | [19] |
| CMI4.158 | C1MI.4 | 1583 | LK931490 | [19] |
| CMI5.170 | C1MI.5.1 | 1706 | LT15554 | [27] |
| CMI5.240 | C1MI.5.2 | 2406 | LT15554 | [27] |
| HCBI3.108 | C1HB.3 | 1086 | LK931495 | [17] |
| HCBI4.296 | C1HB.4 | 2958 | LK931496 | [17] |
| HCBI5.173 | C1HB.5 | 1723 | LK931497 | [17] |
| HCBI6.252 | C1HB.6.1 | 2522 | LK931493 | [19] |
| HCBI6.159 | C1HB.6.2 | 1591 | LK931494 | [19] |
|  | | | | |
| **New isolates** | | | | |
| C1MI.15M.1 |  | 2040 | LR215494 | This study |
| C1MI.15M.2 |  | 2041 | LR215495 |  |
| C1MI.9M.1 |  | 1935 | LR215496 |  |
| C1MI.9M.2 |  | 1934 | LR215497 |  |
| C1MI.3M.1 |  | 1767 | LR215499 |  |
| C1MIs.3M |  | 461 | LR215498 |  |
|  | | | | |
| **Previous BMMF2 isolates** | | | | |
| HCBI1.225 | C2HB1 | 2251 | LK931499 | [17] |
| HCBI2.170 | C2HB2 | 1407 | LK931500 | [17] |
| HCBI7.228 | C2HB7 | 2280 | LK931498 | [17] |

Group1 – BMMF1 (Bovine Meat and Milk Factor)

Group 2 – BMMF2

HCBI1 - healthy cattle blood isolate 1

C2HB1 - cattle group2 healthy bovine isolate 1

MSBI1.176 – multiple sclerosis brain isolate 1 (size 1765nt)

H1MSB1 – human group 1 multiple sclerosis brain isolate 1

CMI1.252 – cow milk isolate 1 (size ca 2520nt)

C1MI.1 – cattle group 1 milk isolate 1

C1MI.15M.1 – cattle group 1 milk isolate, sample 15, primer M, isolate 1
